# EXERTIONAL COMPARTMENT SYNDROME AND MEDIAN NERVE COMPRESSION CAUSED BY A REVERSED PALMARIS LONGUS

**DOI:** 10.2340/jrm.v58.46187

**Published:** 2026-07-02

**Authors:** Diogo FERREIRA, João GOMES, Hugo AMORIM

**Affiliations:** Physical Medicine and Rehabilitation Department, São João Hospital, Alameda Professor Hernâni Monteiro 4200-319 Porto, Portugal

The palmaris longus muscle (PL) is a muscle located superficially, in the anterior aspect of the forearm, right beneath the skin and subcutaneous fat and between the flexor carpi radialis and flexor carpi ulnaris muscles. It has a spindle-shaped appearance, sharing a common origin site with the pronator teres, flexor carpi ulnaris, flexor digitorum superficialis, and flexor carpi radialis muscles, called the common flexor tendon, at the medial epicondyle of the humerus and inserts, generally, at the palmar aponeurosis (1–4). Despite being the target of a wide variety, its structure can generally be described as follows: a proximal section consisting of a tendon, a middle section consisting of a muscle belly, and a distal section consisting of a long thin tendon. The PL acts as an accessory wrist flexor (1, 3, 5).

The PL presents with a variant in approximately 20% of the population (6). Its most common anatomical variant is unilateral agenesis (1, 4–6). Other variants include bilateral agenesis, triple-headed palmaris longus, accessory palmaris longus, bifid palmaris longus, and one of the most infrequent anatomical variants, a reversed palmaris longus (RPL) (3–5, 7). RPL was first documented and anatomically described in 1916 (8). The latter is described as a variant where the muscle belly is located distally, near or at the wrist.

The PL, more specifically its distal tendon, has a close anatomical relationship with the carpal tunnel, running superficially to the flexor retinaculum (1, 2, 4). As mentioned before, its structure varies widely and the anatomical variations of this muscle represent a potential site of pain and peripheral nerve entrapment at the wrist (1, 2, 4–7).

## CASE DESCRIPTION

A 40-year-old female seamstress presented to our physical medicine and rehabilitation outpatient clinic with a 6-month history of left wrist pain and swelling. Initially, the symptoms were caused by prolonged working hours and lasted for several hours afterwards. The pain progressively increased in frequency, ultimately impairing low-effort tasks such as computer use, and the swelling had slowly increased over this period of time. There was an associated tingling sensation, described in the median nerve distribution, which worsened during manual labour. The patient had no significant medical history. Physical examination revealed a visible swelling in the distal volar aspect of the left forearm and wrist (Fig. 1). Resisted wrist flexion elicited pain. Median nerve examination revealed a positive Phalen’s sign, with no significant sensory or motor deficits. Examination of the right wrist was unremarkable. Ultrasound examination was performed, revealing the distal position of the muscle belly of the palmaris longus in the forearm. Magnetic resonance imaging (MRI) of the left wrist was subsequently requested, which demonstrated an extension of this muscle belly into the proximal region of the carpal tunnel, confirming the diagnosis of an RPL (Fig. 2). Electromyography (EMG) of the left upper limb showed no abnormalities.

**Fig. 1:**
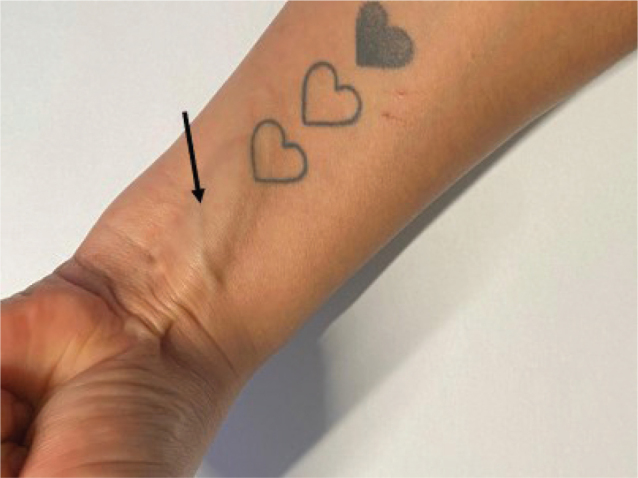
Visible swelling in the distal volar forearm on physical examination (arrow).

**Fig. 2:**
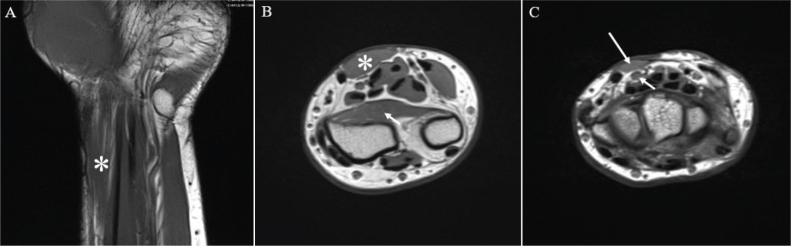
(A) Coronal T1-weighted MRI of the left distal forearm and wrist showing a hypertrophied muscle belly of a reversed palmaris longus (asterisk) located superficially in the distal forearm. (B) Axial T1-weighted MRI at the distal forearm level demonstrating the muscle belly of the reversed palmaris longus (asterisk) located superficially, with the pronator quadratus muscle (arrow) visible in its typical deep position adjacent to the distal radius, serving as an anatomical reference for distal forearm level. (C) Axial T1-weighted MRI at the proximal third of the carpal tunnel showing the muscle belly of the reversed palmaris longus (long arrow) positioned superficially and in close anatomical proximity to the median nerve (short arrow).

Initial management consisted of a period of rest, ice application, and a course of topical and oral non-steroidal anti-inflammatory drugs (NSAIDs). The patient then underwent a rehabilitation programme focused on improving pain and hand function, which included stretching exercises for the intrinsic and extrinsic muscles of the hand and wrist, transcutaneous electrical nerve stimulation (TENS) in continuous mode (Mode C), and myorelaxant massage therapy with gentle deep tissue and kneading techniques applied to the forearm musculature to relieve muscle tension and promote relaxation. Fine motor training exercises were also performed to improve dexterity, coordination, and overall functional use of the hand. Symptoms subsided partially, with pain relief and reduction of swelling, particularly those associated with work-related activities. The patient was referred for an orthopaedic consultation, where surgical removal of the muscle is being considered.

## DISCUSSION

The currently available literature reveals that anatomical variants of the PL are a rare cause of wrist pain and carpal tunnel syndrome.

Our patient presented with painful swelling of the left wrist and effort-related paraesthesia in the median nerve distribution. In the absence of traumatic, degenerative, or inflammatory causes and in the presence of a gradually growing mass, rarer causes, including tumours, must be considered (9). Additionally, repetitive movements of the hand and wrist can lead to hypertrophy of the RPL. The RPL is enclosed by a fascial sheath, and repetitive muscle activity often results in increased pressure within the anterior compartment of the forearm, thereby compromising local blood circulation. Consequently, the RPL has been associated with exertional compartment syndrome, which manifests as painful swelling at the distal forearm and wrist (4–7). A complete radiological work-up should be performed. Despite not being carried out in our case, plain radiography is the first step, primarily aimed at ruling out bony abnormalities such as fractures or bone tumours. If radiographs are unremarkable, ultrasound should be performed prior to proceeding with MRI. A superficial isoechoic structure relative to surrounding muscles, located in the distal forearm and wrist region, is suggestive of an anatomical variant of the PL, namely an RPL. MRI, the most accurate modality for soft tissue evaluation, ultimately confirms the diagnosis, providing a more precise assessment of the muscle belly’s morphology and topography. T1- and T2-weighted MRI images typically show a mass in the distal forearm to wrist region with signal (9). This is particularly important in the event that surgical intervention becomes necessary in the future.

In a PL without anatomical variation, its long distal tendon runs superficially to the carpal tunnel, where the median nerve is located (1, 2, 4). In cases of RPL, where a frequently hypertrophied muscle belly lies superficial to the transverse carpal ligament and compresses the median nerve, typical symptoms of carpal tunnel syndrome may occur (2, 5–7). In our patient, paraesthesias in the median nerve distribution were intermittent and worsened particularly with manual work. There were no other associated neurological deficits, such as motor weakness or sensory loss. Given the suspicion of associated carpal tunnel syndrome, an EMG was requested, which returned without abnormalities. The diagnosis of carpal tunnel syndrome is primarily clinical. EMG in these cases has a sensitivity of approximately 77%, with false negatives occurring in up to 35% of cases (10), particularly in patients with mild forms of the condition, as is the case here.

For symptomatic cases, such as ours, initial management should involve conservative treatment, including relative rest with activity modification, cryotherapy, NSAIDs, a rehabilitation programme aimed at pain relief, improvement of hand function, and fine motor skills, and the use of wrist orthoses. Surgical intervention is reserved for cases that are refractory to conservative measures, consisting of open excision of the RPL (9).

### Conclusion

Our case underscores a rare but clinically relevant cause of wrist pain and median nerve-related symptoms: an RPL. In a middle-aged manual worker with a gradually enlarging volar forearm mass, exertional pain, and intermittent paraesthesia in the median nerve distribution, targeted imaging, including ultrasound followed by MRI, was crucial to establish the anatomic diagnosis and to delineate its relationship to the carpal tunnel. The normal electrodiagnostic study highlights that carpal tunnel syndrome associated with an RPL may be intermittent or mild and therefore may not be detected on EMG. Consistent with best practice, a stepwise approach beginning with conservative measures led to partial symptomatic improvement. Surgical excision remains an option for persistent, function-limiting symptoms.
